# Periluminal Distribution of HIV-Binding Target Cells and Gp340 in the Oral, Cervical and Sigmoid/Rectal Mucosae: A Mapping Study

**DOI:** 10.1371/journal.pone.0132942

**Published:** 2015-07-14

**Authors:** Mariia Patyka, Daniel Malamud, Drew Weissman, William R. Abrams, Zoya Kurago

**Affiliations:** 1 Faculty of Medicine, Division of Experimental Medicine, McGill University, Montreal, Quebec, Canada; 2 NYU College of Dentistry, Department of Basic Sciences, HIV/AIDS Research Program (HARP), New York, New York, United States of America; 3 NYU School of Medicine, Infectious Disease, New York, New York, United States of America; 4 Medicine (Infectious Disease), Perelman School of Medicine, University of Pennsylvania, Philadelphia, Pennsylvania, United States of America; 5 Oral Health and Diagnostic Sciences, College of Dental Medicine, Georgia Regents University, Augusta, Georgia, United States of America; Deutsches Primatenzentrum GmbH - Leibniz-Institut fur Primatenforschung, GERMANY

## Abstract

Studies have shown that the transmission of HIV is most likely to occur via rectal or vaginal routes, and rarely through oral exposure. However, the mechanisms of virus entry at mucosal surfaces remain incompletely understood. Prophylactic strategies against HIV infection may be attainable once gaps in current knowledge are filled. To address these gaps, we evaluated essentially normal epithelial surfaces and mapped the periluminal distribution of CD4^+^ HIV target cells, including T cells and antigen-presenting cells, and an HIV-binding molecule gp340 that can be expressed by epithelial cells in secreted and cell-associated forms. Immunohistochemistry for CD4, CD16, CD3, CD1a and gp340 in human oral, rectal/sigmoid and cervical mucosal samples from HIV-negative subjects demonstrated that periluminal HIV target cells were more prevalent at rectal/sigmoid and endocervical surfaces lined by simple columnar epithelium, than at oral and ectocervical surfaces covered by multilayered stratified squamous epithelium (p<0.001). gp340 expression patterns at these sites were also distinct and strong in oral minor salivary gland acini and ducts, including ductal saliva, in individual rectum/sigmoid and endocervix periluminar columnar cells, and in ectocervix squamous cells. Only weak expression was noted in the oral non-ductal squamous epithelium. We conclude that periluminal HIV target cells, together with periluminal epithelial cell-associated gp340 appear to be most accessible for HIV transmission at rectal/sigmoid and endocervical surfaces. Our data help define vulnerable structural features of mucosal sites exposed to HIV.

## Introduction

Infections by HIV remain a major global public health problem. Anti-retroviral treatment (ART) has provided a means to control the progression of the disease, but treatment is expensive and a cure remains elusive. As with other infections, effective prevention is critical to controlling the spread of disease. Efforts to develop effective prevention are ongoing and prophylactic strategies will be improved once the pathways of HIV entry at mucosal surfaces are better understood. The majority of infections worldwide occur through vaginal and rectal intercourse, while infections in adults following the exposure of the oral mucosa to HIV are rare [1–3]. Saliva is considered a potential contributor to the apparent resistance to infection via the oral cavity, as it contains a variety of factors that can bind, opsonize, and/or neutralize bacteria and HIV-1 [2].

CD4 is the primary receptor for HIV-1 [4] and is expressed at high levels on a subset of T cells and at low levels on monocytes, macrophages, dendritic cells (DC), and microglia [5], thus making these cell populations good HIV targets. HIV entry into CD4^+^ target cells requires binding to co-receptors, typically the chemokine receptors CCR5 or CXCR4 [6]. Tissue-associated macrophages and DC express both co-receptors and are highly susceptible to infection and viral replication [7];[8]. Moreover, mucosal DC associated with columnar epithelium in the gut were shown to extend and return processes containing HIV between columnar epithelial cells [9], and DC contribute to viral dissemination into CD4^+^ T cells [10]. Because rapid entry into a target cell protects the virus from host defenses, access to CD4^+^ target cells at mucosal sites is likely a critical step in HIV infection, and consequently, differential access among mucosal sites may impact susceptibility to infection.

Other cell surface and soluble molecules are also known to bind to HIV, which may either aid or prevent HIV entry into target cells. Among soluble host molecules of particular interest is gp340, also known as salivary agglutinin, a 340kDa glycoprotein member of the DMBT1/scavenger receptor cysteine-rich (SRCR) super family involved in innate immune defenses. In the oral cavity, salivary glands secrete high levels of soluble gp340 [2, 11]. Soluble gp340 has been shown to bind to the stem of the gp120 V3 loop, which is postulated to interfere with HIV entry [11–13]. In contrast, vaginal and cervical epithelial cells express gp340 bound to the cell surface, which may facilitate the transmission of virus to susceptible target cells [12, 14, 15]. Because soluble and cell surface-bound gp340 have opposite roles in HIV infection, it is important to determine where and in what form gp340 is expressed at the three mucosal sites of transmission (oral, vaginal / cervical, rectal) and its anatomical relationship to CD4^+^ target cells at these sites.

Furthermore, there are mucosal structure considerations that could impact HIV entry. The oral cavity and vagina/ectocervix (the cervical surface exposed to the vaginal cavity) are lined by multi-layered stratified squamous epithelium, while the endocervix (the cervical canal) and colon/rectum are lined by a thin layer of simple columnar epithelium. Multilayered stratified squamous epithelium serves as a strong barrier to both external and internal substances and withstands mechanical trauma, which is in contrast to the permeable and relatively fragile, one cell layer-thick columnar epithelium designed for secretion and absorption.

We hypothesized that besides the structural epithelial differences at the three mucosal sites of HIV entry, i.e. distal colon/rectum, cervical mucosa (ecto- and endocervix) and oral mucosa, the distribution of CD4^+^ HIV target cells and/or of gp340 may be distinct. In this study, we used immunohistochemistry with archival human samples to map the distribution of CD4^+^ cells, including T cells and antigen presenting cells (APC, i.e. myeloid dendritic cells, monocytes and macrophages) and gp340 throughout the surface epithelia at the indicated anatomic sites. We found that, consistent with our hypothesis, the single layer columnar epithelium of the colon/rectum and endocervix contained gp340-positive epithelial cells at the luminal surfaces, mingling with HIV target cells. In contrast, CD4^+^ cells were rarely seen at the luminal surfaces of stratified squamous epithelium in the oral cavity or ectocervix. Most of the oral squamous epithelium showed weak gp340 staining, while oral minor salivary glands, their ductal epithelium and duct contents were strongly gp340-positive. However, the ectocervical squamous epithelium often showed intense gp340 labeling throughout.

## Materials and Methods

### Tissue samples

The NYU Medical Center IRB and the NYU University Committee on Activities Involving Human Subjects (exempt protocols) specifically approved the entire study. Archival, de-identified samples were used. All tissue samples had been fixed in standard 10% phosphate-buffered formalin (Thermo Fisher Scientific, Fremont, CA) and processed by uniform standard medical/pathology laboratory procedures using automated tissue processing. Eight cervical, eleven rectal and ten sigmoid colon samples were obtained from HIV-seronegative individuals after surgery for tumors, and the adjacent non-tumor tissues removed as part of the surgical procedure were used for our studies. Ten oral mucosal samples from HIV-negative subjects included biopsy specimens of non-specific mucositis or keratosis with adjacent normal tissues; oral samples were obtained from the archives of the NYU College of Dentistry Diagnostic Pathology Laboratory, which were also processed by standard medical/pathology laboratory procedures. Although data on the presence of sexually transmitted infections in the study populations were not available, this information was not essential, because the analysis was confined to morphologically normal mucosal surfaces showing little to no inflammation. The Histopathology Core of NYU BioRepository Center (BRC) provided serial sections. Serial (i.e., back-to-back) 5μm sections, were used in order to evaluate potential co-localization of molecules and cells within 10–50 μm, using single-color immunohistochemistry for best resolution of staining with minimal non-specific background.

### Antibodies

Mouse monoclonal anti-gp340 IgG (Mab143 [16], mouse anti-CD1a IgG (clone O10) (Thermo Fisher Scientific), rabbit anti-CD3 (SP7) (Thermo Fisher Scientific), mouse anti-CD4 IgG (clone 4B12) (Thermo Fisher Scientific), mouse anti-CD16 IgG (clone 2H7) (AbD Serotec, Raleigh, NC) were used. Negative controls were non-specific, isotype and concentration-matched mouse IgG (Southern Biotech, Birmingham, AL) and polyclonal rabbit antibodies (CD3 negative control). Primary antibody binding to tissue targets was detected using the LP Value HRP-DAB Detection kit (Thermo Fisher Scientific). All reagents were titrated and the commercial LP Value HRP-DAB Detection kit was used according to manufacturers’ instructions.

### Immunohistochemistry (IHC)

Standard single-color IHC was performed as described previously [17]. Briefly, 5 μm sections of formalin-fixed, paraffin-embedded tissue were deparaffinized and rehydrated by standard methods. Heat-induced epitope retrieval was performed in a 97°C water bath for 20 min in citrate buffer, pH 6.0 (for anti-CD1a, anti-CD3, anti-CD4, anti-CD16, anti-gp340 and their IgG negative controls), and the sections were re-equilibrated in PBS. Endogenous peroxidase and non-specific antibody binding were blocked (Ultra-V and Hydrogen Peroxide, LP Value kit), and then sections were incubated with respective primary antibodies at room temperature (30 min) or at 4°C (overnight), followed by detection with Enhancer, horseradish peroxidase (HRP)-Polymer, and diaminobenzidine tetrahydrochloride (DAB) substrate, all according to the manufacturer’s instructions (Fisher Scientific). All incubations, except non-specific protein binding (Ultra-V, LP Value Detection kit) block, were separated by washing with PBS. Coverslips were applied with aqueous mounting medium (Thermo Fisher Scientific) and photomicrographs were obtained (Olympus Corp., Tokyo, Japan). Each IHC run included sections stained with non-specific antibodies matched for species, isotype and concentration with the specific primary antibodies (negative controls).

### Data analysis

IHC-stained slides were digitally photographed at 100x, 200x and 400x magnification. Intact epithelial surfaces were evaluated. Non-overlapping, sequential images were acquired using the same orientation of the epithelial surfaces parallel to the image border, ensuring consistent sampling of the epithelial surfaces for all sections. Marker-positive cells were counted manually at the 200x magnification, recording each field separately, and the number of fields evaluated for each group is indicated in Table 1. The distribution of signal-positive cells (including CD3^+^, CD4^+^, CD16^+^, CD1a^+^, GP340^+^) throughout the epithelial surfaces was documented. The intraepithelial location of each marker-positive cell was determined on the basis of epithelial and connective tissue morphology, as the cell shape, nuclear polarization and basement membrane zone are identifiable in both IHC and H&E-stained sections (examples are included in S5 Fig). CD16^+^ neutrophils identifiable by lobular nuclei were excluded from analysis. Using the images, we quantified the distribution of CD4^+^ and CD16^+^ mononuclear cells in the stratified squamous epithelia at each of the sites, including the cells in the periluminal layer of squamous epithelia (oral and ectocervix) and compared to those in the single-layered columnar epithelia (rectum/sigmoid and endocervix). For the purposes of consistency, intestinal crypts, endocervical tunnels and clefts were excluded from cell counts, because oral mucosa is devoid of such structures. Differences between groups were evaluated using the Mann-Whitney test, because the data were skewed.

**Table 1:** Distribution of CD4+ and CD16+ cells within the stratified squamous (oral mucosa and ectocervix) and columnar (endocervix and rectum/sigmoid) epithelia.

			Periluminal Epithelial Layer		
Sites	Cell Types	Cell Counts (Total)	Positive Cell Count	% Positive Cells	# Fields
**Oral mucosa**	CD4^+^	730	6	0.8	34
	CD16^+^	127	3	2.4	20
**Ectocervix**	CD4^+^	721	11	1.5	29
	CD16^+^	276	11	4.0	10
**Endocervix**	CD4^+^	57	57	100.0	12
	CD16^+^	115	115	100.0	12
**Rectum/sigmoid**	CD4^+^	88	88	100.0	33
	CD16^+^	136	136	100.0	26

## Results

### Distribution of CD4^+^ and CD16^+^ cells in the stratified squamous oral and ectocervical epithelia, and in columnar epithelia of the endocervix and rectum

**Fig 1:**
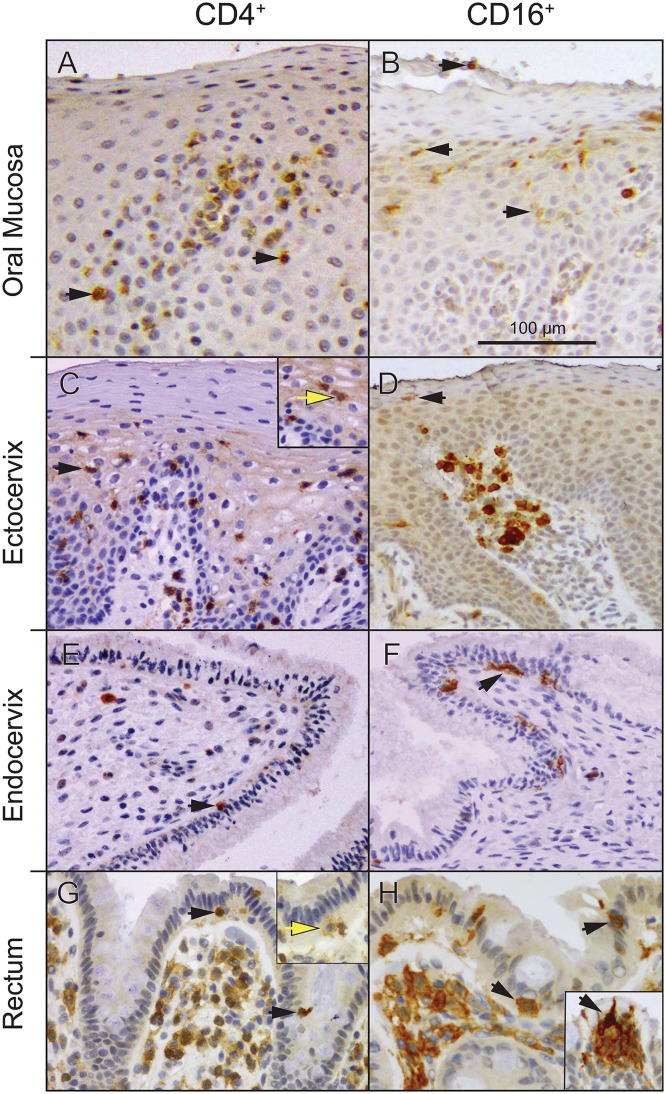
Distribution of CD4^+^ and CD16^+^ cells in squamous epithelia of the oral and ectocervical mucosae and in simple columnar epithelia of the rectum/sigmoid and endocervix. Tissue sections were stained by standard IHC for CD4 (Panels **A,C,E,G**) or CD16 (Panels **B,D,F,H**), as described in Materials and Methods. Representative images are shown. Receptor-positive cells appear brown and cell nuclei appear blue. Arrows indicate a few examples of positive cells located within the epithelium. Small round CD4^bright^ cells (black arrows) are T cells, while antigen-presenting cells (APC) are larger and CD4^dim^ (yellow arrows). Note that CD4^+^ cells are typically present several cell layers deep within the stratified squamous epithelium and lamina propria of oral (**A**) and ectocervical mucosae (**C**), similar to CD16^+^ APC (**B** and **D**, respectively). Small round CD4^bright^ lymphocytes, CD4^dim^ APC and CD16^+^ APC are also present within the simple columnar epithelia (**E-H**). Note the horizontal orientation of CD16^+^ APC at the base of columnar cells in the endocervix (**F**). Also note that some CD16^+^ APC in the rectal epithelium are oriented vertically with dendritic processes reaching the luminal surface (insert, **H**). Bar = 100 μm.

CD4^+^ T cells express high levels of CD4, while monocytes, macrophages and myeloid dendritic cells express CD4 at low levels [5]. All of these populations are found in quiescent mucosal tissues and at sites of inflammation. Representative examples of CD4^+^ cell distribution in the squamous epithelia of oral and ectocervical mucosae, and in simple columnar epithelia of the endocervical and sigmoid/rectal mucosae are shown in Fig 1. A more intense CD4 labeling was noted on small round lymphocytes, consistent with T cells. This was further supported by comparable CD3 expression on small lymphocytes at the same sites (S1 Fig). Large irregularly-shaped cells, some of which had dendritic processes, showed weak CD4 labeling, consistent with monocytes/macrophages and dendritic cells (APC). Because of low signal to noise ratio of CD4 labeling on APC, detection of these cells in epithelia was difficult. Therefore, the presence of APC in the same distribution was confirmed by staining for CD16 (Fig 1), because mucosal and inflammation-associated monocytes-macrophages and DC express CD16. In addition, some samples were stained for CD1a (S1 Fig), as DC also express CD1a, particularly when associated with surface epithelium [18].

The epithelial distribution of CD4^+^ cells varied depending upon the site and cell subset. Specifically, CD4^bright^ small lymphocytes were present in both squamous and columnar epithelia, as were the large CD4^dim^ cells. Within oral and ectocervical squamous epithelium, CD4^+^ cells were more numerous at sites of apparent mild inflammation, i.e. in the context of inflammatory cell infiltrates in the underlying lamina propria. The vast majority of the intraepithelial CD4^+^ cells were seen in the deeper half of the epithelial thickness, several cell layers away from the luminal surface, while in the ectocervical and oral squamous epithelia, periluminal CD4^+^ cells were rare (Fig 1A–1D, Table 1). The results of statistical analysis of cell counts in the periluminal layers are shown and discussed below.

Intraepithelial CD16^+^ cells appeared to be less common than CD4^+^ cells in the same squamous epithelial sites (Table 1). Few intraepithelial CD16^+^ cells were found in the periluminal layer of the squamous epithelia lining the ectocervix and the oral mucosa (Table 1).

Both CD4^+^ and CD16^+^ cells were identified within single cell-layered endocervical and rectal/sigmoid epithelia (Fig 1E–1H). CD4^bright^ and CD4^dim^ cells were found at the base of the columnar cells near the basement membrane or at the columnar cell apex (Fig 1 and S3–S5 Figs).

At both columnar epithelial sites, large mononuclear CD16^+^ APC were easily detectable and were often seen oriented horizontally along the base of the columnar epithelial cells (Fig 1F, arrow; S5D Fig). In the bowel lining, but not in the endocervix, these cells were also found oriented vertically throughout the epithelial thickness from the base to the apex (Fig 1H). However, the amount of endocervical tissue available for CD16 analysis was relatively small.

Clearly, all CD4^+^ and CD16^+^ cells entering the single-layered simple columnar epithelia were periluminal, while the vast majority of such cells in squamous epithelia remained deep, several epithelial cell layers away from the lumen (Table 1; Fig 1, S3–S5 Figs).

### Distribution of cell-associated gp340 in the epithelia of oral, cervical and rectal/sigmoid mucosa

**Fig 2:**
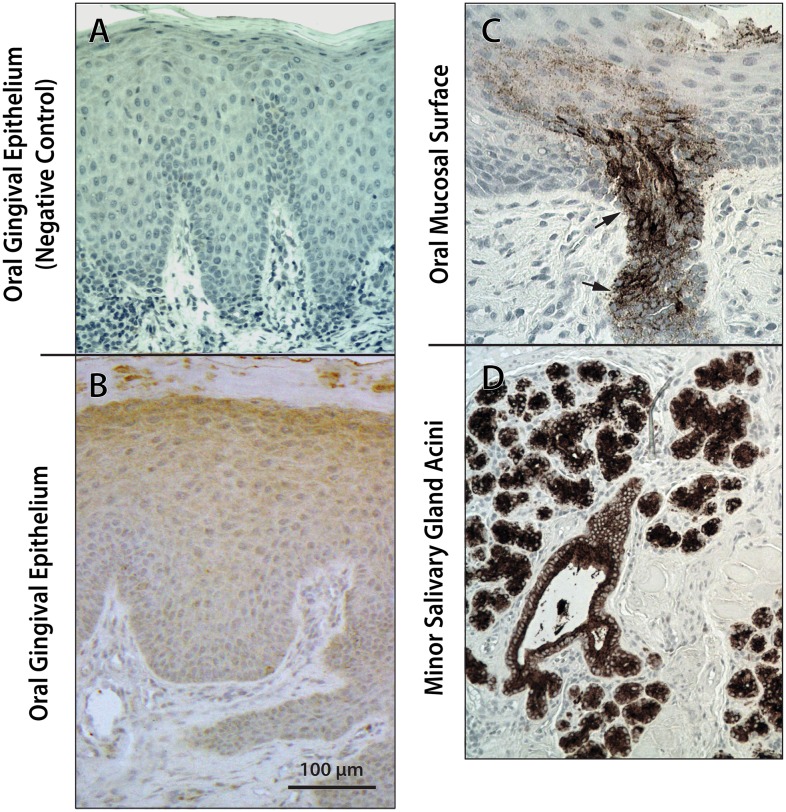
Expression of gp340 in keratinized squamous epithelia of the oral gingival mucosa (B) and non-keratinized epithelium of the oral mucosa with associated salivary gland acini and ducts (C, D). Tissue sections were stained by standard IHC for gp340 using the monoclonal antibody 143.D4 IgG, as described in Materials and Methods. Representative images are shown. Brown stained areas are gp340 and cell nuclei appear blue. Negative control is shown in **(A)**. Note diffuse granular gp340 staining in squamous cells and patchy gp340 staining in the surface parakeratin of oral gingival epithelium **(B)**. A minor salivary gland duct **(C)** arising from the underlying gland **(D)** merges with the surface non-keratinized stratified squamous epithelium and shows intense brown granular gp340 staining of the ductal cells (arrows). The minor salivary gland acini with a central duct and saliva inside the duct **(C)** also show intense gp340 staining.

**Fig 3:**
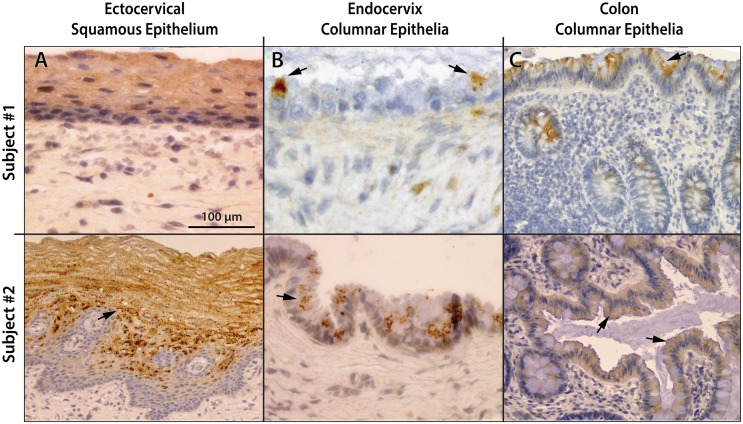
Expression of gp340 in the ectocervix, endocervix, and colon. Tissue sections were stained by standard IHC for gp340 using the monoclonal antibody 143 IgG, as described in Materials and Methods. Representative images are shown. Brown stained areas are gp340 and cell nuclei appear blue. Sections of cervix and colon from two different subjects show diffuse granular staining of epithelial cell-associated gp340 throughout the ectocervical squamous epithelium **(A)**. The columnar epithelium of the endocervix **(B)** and colon **(C)** shows single-cell (Subject #1, Endocervix) and patchy (multiple adjacent cells, Subject #2, Endocervix) granular staining of the columnar epithelia in the endocervix and colon. Several examples of gp340^+^ cells are indicated with arrows. Negative controls are shown in Fig 2A (squamous epithelium) and S2A Fig (columnar epithelium).

The expression of gp340 in the epithelia varied depending upon the anatomic site as well as the individual sample. Representative images are included in Figs 2 and 3, as well as S2–S4 Figs. Throughout the various epithelia, gp340-specific staining was consistently granular, irrespective of the labeling intensity. Gp340 was present in the keratinocytes of the differentiated spinous layer in the stratified squamous epithelia of the ectocervix and the oral mucosa. Labeling intensity varied in both oral and ectocervical epithelia. However, in contrast to often intense keratinocyte staining throughout the ectocervical spinous layer (Fig 3, S4B Fig), the most intense gp340 staining in the oral squamous epithelium was confined to foci where minor salivary gland ducts merged with the surface (Fig 2C). On the other hand, samples from the gingiva (a site devoid of minor salivary glands, Fig 2B) showed mild diffuse staining of the spinous layer and sometimes of the parakeratin, but not focal intense staining as seen in ductal cells merging with the surface epithelium.

The columnar epithelia of the endocervix and of the rectum/sigmoid showed similar patches of gp340 expression in columnar epithelial cells, including the mucosal surface, the intestinal crypts, and the endocervical tunnels and clefts, ranging from undetectable to intense, without apparent staining of mucous goblet cells (Fig 3, S2, S3 and S4D Figs). The expression of gp340 and the presence of intraepithelial HIV target cells often coincided (S3 and S4 Figs). Paired cervix and colon specimens from the same individuals showed variable intensity of gp340 staining at the three corresponding epithelial surfaces, suggesting that local factors likely influence gp340 expression (Fig 3).

### Statistical analysis

**Fig 4:**
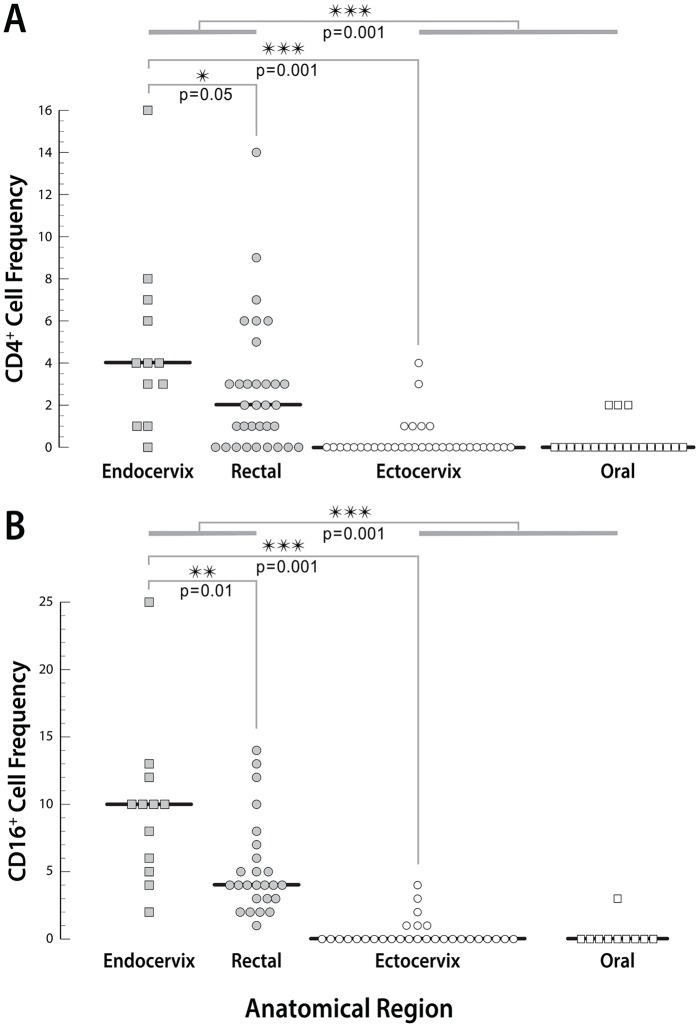
Comparison of periluminal CD4^+^ (A) and CD16^+^ (B) counts at all sites. Stained and photographed sections (200x) were used to record the CD4^+^ and CD16^+^ cells, respectively, in the periluminal layer of squamous epithelia and in single-layer simple columnar epithelia. Charts show respective cell counts per field by region, each data point is one field. Mann Whitney tests were used to compare the columnar and squamous epithelial groups, as well as individual sites and resulting p values are indicated on the graph.

The luminal mucosal surfaces are directly exposed to the virus, which makes the periluminal cell layer critical. We further analyzed HIV target cells in the periluminal layers of the four anatomical sites (Fig 4). Statistical analysis of cell counts in periluminal epithelial layers revealed that CD4^+^ cell numbers were highest in the endocervix, followed by rectum/sigmoid and lowest in oral and ectocervical squamous epithelia, and the difference between squamous and columnar epithelia was significant (p< 0.001). While there was no difference in periluminal CD4^+^ counts between ectocervical and oral squamous epithelia, CD4^+^ cells were significantly more common in the endocervical epithelium *vs* the rectal lining (p = 0.05). Similarly, CD16^+^ cells were most frequent in the endocervical columnar epithelium, followed by sigmoid/rectal lining, and negligible in periluminal layers of both squamous regions, with a significant difference between columnar and squamous sites (p < 0.001). Again, there was no difference in CD16^+^ counts in the periluminal layers of the two squamous regions, but the endocervical lining had significantly more CD16^+^ cells than the rectal/sigmoid epithelium (p = 0.01).

## Discussion

The periluminal, intraepithelial distribution of HIV target cells and of an alternative HIV-binding molecule gp340 at all the mucosal sites exposed to HIV transmission were characterized and compared, because the epithelial periluminal surfaces are critical for initial binding of the virus for transmission. On the basis of our mapping study for CD4, CD16 and gp340, the essentially normal mucosal surfaces can be ranked from the most HIV-resistant oral mucosa, followed by ectocervical mucosa, and finally by the least resistant endocervix and rectum, an interpretation in overall agreement with epidemiological data. HIV transmission studies show that in adults, rectal transmission occurs in 1:20–1:300 infections per exposure, vaginal transmission is estimated at between 1:200 to 1:2000 exposures [19], and HIV transmission by fellatio is rare [1]. These rates are influenced by a variety of factors, including integrity of the mucosa and other HIV-binding molecules, discussed below.

Our data also suggest that the endocervical lining may be more susceptible to infection than rectal mucosa due to significantly more frequent periluminal presence of HIV target cells. Yet, the rate of vaginal HIV transmission is much lower than rectal [19]. The relatively low vaginal transmission could be due to a smaller surface area covered by columnar epithelium and accessible to HIV. Most of the rectum is lined by simple columnar epithelium, the rectum is 10-15cm long and 2–5 cm in diameter [20], resulting in a large surface area. On the other hand, the cervix is only 3–4 cm long with at most 6–8 mm in endocervical diameter [21], while the vaginal cavity, including the ectocervix in most cases, is surfaced by non-keratinized stratified squamous epithelium with very few HIV target cells in the periluminal layer. The endocervical columnar epithelium can extend out onto the ectocervix in the vaginal cavity, called the ectropion [21], which has been shown to increase the risk of acquiring HIV infection by women who are partners of HIV-positive men [22]. The ectropion is seen in many women during early childbearing age, may cover most of the ectocervix and even extend into the vaginal fornix, increasing with pregnancy and the use of combined contraceptives [23, 24]. With advancing age and in the absence of hormones, the squamocolumnar junction ends up deep in the endocervix due to squamous metaplasia [23, 25], shrinking the surfaces covered by columnar epithelium and likely further contributing to low vaginal HIV transmission rates.

HIV-1 entry into CD4^+^ cells is mediated by co-receptors CCR5 (R5 viruses) and CXCR4 (X4 viruses) [26], and CCR5 is dominant over CXCR4 in HIV transmission from person to person and early in infection [8]. HIV binding to the co-receptors requires initial binding to CD4, which is followed by specific conformational changes permissive to co-receptor engagement [26], [27]. Recent studies using multi-color fluorescence demonstrated numerous CCR5^+^ T cells, monocytes/macrophages and DC in the distal large intestine, especially prominent in the rectum [28], as well as in the cervix [29]. In these studies, the specific location of the stained cells relative to the lumen was not addressed, though most of the cells appeared to be in the lamina propria and possibly, the crypts. It is reasonable to propose that periluminal CD4^+^ cells, with or without the help of gp340 or other alternative binding molecules, could bind the virus, and become targets of infection in the presence of CCR5. In the absence of their own co-receptor, CD4^+^ cells could transfer the virus to the CD4^+^CCR5^+^ cells elsewhere in the mucosa. Consistent with this notion, DC processes can extend between columnar epithelial cells in the colonic mucosa and take up HIV [9], and DC are well known to disseminate the virus to T cells [10].

Mucosal damage due to abrasions, erosions and inflammation, or thinning in response to hormonal therapy, have all been shown to increase transmission (reviewed in [19]). Moreover, simple columnar epithelium is quite fragile and more prone to damage relative to stratified squamous epithelium. Sexually transmitted diseases causing genital mucosal inflammation and damage increase HIV transmission 2-11-fold [30]. These increases are likely due to multiple factors, including induction or up-regulation of the expression of HIV co-receptors and their ligands, increase in the numbers of HIV target cells entering the epithelium at sites of inflammation [19, 30], as well as fragility and permeability of inflamed mucosa with loss of epithelial surface integrity that exposes the connective tissue containing increased infiltrates HIV target cells attracted to the inflamed tissues [31]. For example, chemoattractants specific for CCR5 are expressed at sites of inflammation, and Th1-type of inflammation is known to increase CCR5 expression levels [32]. HIV can also induce activation of toll-like receptors TLR2 and TLR4 [33], which contributes to inflammation. It is possible that expression of one or more of the alternative HIV-binding receptors described below, are altered during inflammation, contributing to enhanced transmission of the virus.

Alternative HIV-binding receptors gp340, heparan sulfate proteoglycans (including syndecan-1 CD138), DC-SIGN, DC-SIGNR, langerin, mannose receptor [34], galactosyl ceramide (GalC) and some of its derivatives [35, 36] can affect HIV transmissionIV transmission. While soluble molecules, such as gp340 in saliva, serve neutralizing functions [11]; [12]; [13], cell-associated molecules can facilitate HIV surface binding and transmission. For example, epithelial cells express large amounts of heparan sulfate which sequesters virus particles [37], and transcytosis through primary genital epithelial cells was found to be dependent on syndecan [38]. Many other soluble immune factors are also present in saliva and in rectal lavage fluids. However, saliva was shown to be a significantly more potent inhibitor of HIV infection than rectal fluids, even when saliva was nearly 10-fold less concentrated than rectal lavage fluid (70–80% inhibition at 0.3 μg/ml vs. 40% inhibition at 2 μg/ml respectively) [39]. The specific contributions of individual soluble and cell-bound alternative HIV-binding molecules to HIV transmission have not been quantified.

Among HIV binding molecules, gp340 appears to be important at mucosal surfaces, because it is expressed both on mucosal epithelial cells and in secretions, such as saliva, tears, bronchoalveolar fluid and pancreatic juice [2, 40], and it binds to a variety of microorganisms, including HIV [12, 40]. The highest concentration of secreted gp340 is found in saliva [41], and salivary gp340 was shown to inhibit HIV-1 infection by binding to viral gp120 [42]. Consistent with high levels of gp340 in saliva, we found strong acinar, ductal and intraductal staining for gp340 in oral minor salivary glands. Minor salivary glands are associated with mostly non-keratinized stratified squamous epithelium of the labial and buccal mucosa, floor of mouth, ventral tongue and posterior palate. How accessible the cell-associated gp340 of salivary gland ducts is to HIV binding is not known. Considering the high salivary levels of secreted gp340 and other HIV-binding and inhibiting soluble components, as well as the flow of saliva out of the ducts that are merging with the surface, most of the virus entering the oral cavity is likely neutralized and prevented from binding to gp340-positive epithelial and CD4^+^ target cells. Moreover, periluminal layers routinely slough leaving intact multilayered squamous epithelium behind, and we observed that HIV target cells are rare in the periluminal epithelial layer of normal oral mucosa or even in the top ½ of the epithelial thickness. Much of the oral mucosa (hard palate, dorsal tongue and gingiva) is covered by keratinized squamous epithelium, so these sites are considered even more resistant due to the extra layers of keratin, in addition to other squamous epithelium and saliva properties mentioned above.

The base of the tongue and oropharyngeal mucosa were not evaluated in our study. This site is distinct, as it contains mucosa-associated lymphoid tissue, including the lingual and palatal tonsils. While the surfaces are composed of stratified squamous epithelium, the lymphoid cells, including CD4^+^ T cells, are known to associate with the periluminal surfaces [43], and thus present a site potentially susceptible to HIV transmission. Yet, oral transmission is rare, consistent with saliva-mediated protection (including secreted gp340).

In contrast to the function of secreted product, gp340^+^ primary cells of the ecto- and endocervix, were shown in vitro to bind HIV-1 and facilitate transmission of the virus to CD4^+^ target cells [12, 15, 44]. In simple columnar epithelium, the periluminal location of HIV target cells, as well as their co-localization with gp340^+^ epithelial cells, suggests an advantage for HIV transmission. Although not quantifiable, there was more intense gp340 staining in the ectocervical squamous epithelium than in the oral squamous epithelium without salivary gland ducts, suggesting that ectocervical squamous epithelium may also be more receptive to HIV binding. How this binding would affect HIV transmission in HIV target cell-poor periluminal squamous layers remains to be determined.

Our study showed that cell-associated gp340 expression was not uniform in columnar epithelia at each of the sites. Mucus-producing goblet cells were typically gp340-negative, and gp340-positive cells were either individually scattered, or seen in patches. There was also no obvious correlation between genital and sigmoid/rectal gp340 expression in the same individual. Recent studies indicate that gp340 is inducible, as the activation of the transcription factor Signal Transducer and Activator of Transcription (STAT3) in intestinal epithelial cells induced the expression of DMBT1, the gene encoding gp340 [45]. STAT3 is readily activated in response to several factors produced during inflammation; such as IL-6, IL-10, IL-22, IL-23, IL-27, therefore local inflammation is likely to regulate gp340 expression. STAT3-activating inflammatory factors can be produced by activated APC, including CD16^+^ monocytes, macrophages and DC. Our own attempts to use IHC for evaluation of activated STAT3 and gp340 co-localization were suggestive, but inconclusive (data not shown). However, increase in epithelial gp340 expression in response to inflammation could facilitate HIV transmission, which is consistent with epidemiological studies.

CD4^+^ T cells are the main HIV targets and our study revealed that, in contrast to squamous epithelia, these cells were periluminal in columnar epithelia, and more common in the female genital tract than in the rectum. Similarly, APC were often periluminal in simple columnar epithelia, but in squamous epithelia they were typically in the deep ½ of the epithelial thickness, several cell layers away from the lumen. DC associated with epithelia, also known as Langerhans cells, express HIV-binding receptors langerin and DC-SIGN. HIV uptake via langerin was shown to result in rapid HIV degradation in acidic compartments [46], while HIV binding to DC-SIGN can increase the concentration of the virus on the DC cell surface and help HIV transmission to T cells by facilitating gp120 interaction with CD4 and HIV co-receptors [47, 48]. Moreover, DC-SIGN^+^ blood DC can transmit HIV-1 to T cells [49], while decidual mononuclear cells expressing DC-SIGN, CD16 and other monocyte-dendritic cell markers, were shown to be susceptible to HIV infection followed by replication [50].

### Model

**Fig 5:**
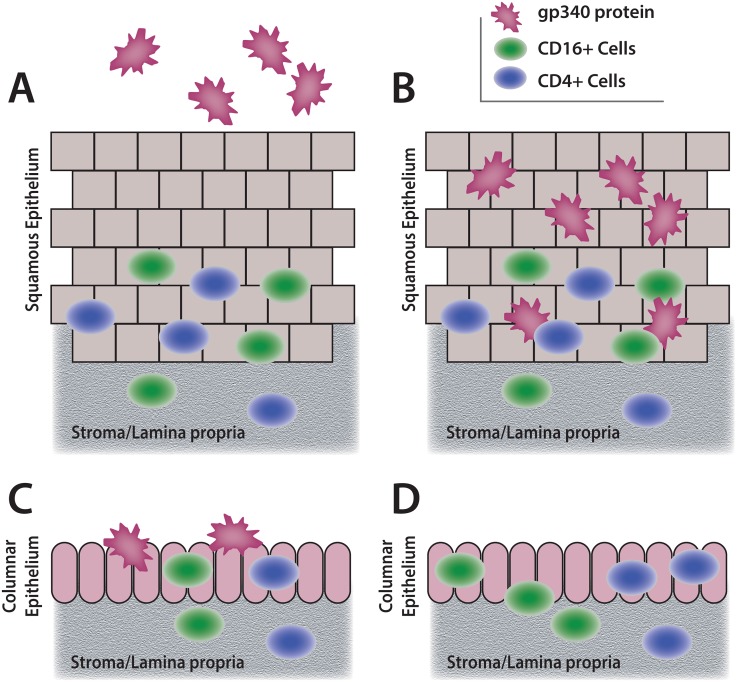
Schematic diagrams summarizing the observed results. The model maps gp340 and HIV target cells at the normal mucosal surface portals. The model proposes that HIV target cells and the epithelial cell-associated alternative HIV-binding molecule gp340 rarely co-localize at the luminal surfaces lined by squamous epithelium, either oral or ectocervical, largely because the target cells typically stay away from the periluminal layer **(A,B)**. Moreover, expression of gp340 in oral squamous epithelium appears to depend largely upon salivary glands, suggesting that it is primarily soluble **(A)**, while the ectocervial squamous epithelium often showed strong gp340 expression throughout **(B)**, without evidence of gp340-secreting glands. In contrast, HIV target cells and cell-associated gp340 frequently, but not always, co-localized periluminally in simple columnar epithelia of the colon/rectum and endocervix **(C, D)**.

The results suggested a model mapping gp340 and HIV target cells at the mucosal surface portals in normal tissues (Fig 5).

In contrast to columnar epithelium (discussed below), HIV target cells rarely associated with the periluminal layer of the stratified squamous epithelium (Fig 5A and 5B). The expression of gp340 in oral squamous epithelium was detectable in the spinous and periluminal layers, but appeared to largely depend on the minor salivary glands. The strongest expression of gp340 concentrated in the glandular acini and ducts, including contents of the ductal lumen, with focal expression in the mucosal surface periluminal cells in non-keratinized squamous epithelium (Fig 5B), consistent with previous reports that in the oral cavity, gp340 exists mainly as a secreted molecule. As the periluminal squamous cells routinely slough into the lumen due to mechanical forces without exposing vulnerable areas, potential for HIV binding to cell-associated gp340 could be of little consequence. Soluble gp340 present in saliva (Fig 5A) could interfere with HIV binding to cell-associated gp340, and epithelial sloughing could also facilitate loss of the rare HIV target cells reaching the periluminal layer. Together, these factors could all contribute to the observed resistance of the oral cavity to HIV infection [51–54]. The diagram in Fig 5B is also representative of our observations in the ectocervical stratified squamous epithelium, which often had strong gp340 expression throughout the spinous and periluminal layers. As the HIV target cells were rare in the periluminal ectocervix, the significance of epithelial cell-associated gp340 for HIV transmission at this site remains to be tested.

The HIV target cells were present at luminal surfaces in simple columnar epithelia of the rectum/sigmoid and endocervix (Fig 5C and 5D), and frequently, the epithelial cell-associated alternative HIV-binding molecule gp340 was detected in the epithelial cells at the same sites (Fig 5C). The implication is that HIV may bind directly to CD4^+^ cells, or first bind to epithelial cell-associated gp340 and then be transferred to the HIV target cells that are in direct contact with gp340-positive cells, with or without epithelial transcytosis. No gp340-positive glands were found in the mucosae of the large intestine or endocervix, and the mucus-producing goblet cells appeared gp340-negative, consistent with reported studies showing low soluble gp340 in rectal and genital fluids.

Our present data suggest a need for investigations of larger sample size, the impact of individual soluble factors, models for comparing the impact of epithelial structure, and contributions of specific intraepithelial cell populations to HIV transmission. Together, our results provide insights into features of mucosal surfaces that help explain epidemiological data on susceptibility of these sites to HIV transmission.

## Supporting Information

S1 Fig Distribution of CD3^+^ T cells and CD1a^+^ DC in the ectocervical, endocervical and rectal epithelia. Tissue sections were stained by standard IHC for CD3 (A, C, E) and CD1a (B, D, F), as described in Materials and Methods. Representative images are shown. Receptor-positive cells appear brown and cell nuclei blue. Arrows indicate a few examples of CD1a-positive cells within the epithelia.
(EPS)

S2 Fig Additional examples of gp340 expression in the colon/rectum. Sections of colon/rectum were stained by IHC for negative control **(A)** or gp340 **(B, C)** as described in Materials and Methods. **A)** Negative control. **B)** High-power detail showing a mucous goblet cell-rich surface and crypt with minimal to none gp340 expression. **C)** High-power detail showing brown granular gp340 staining in columnar epithelial cells, but not in mucous goblet cells.
(EPS)

S3 Fig Examples of co-localization of CD16^+^ cells (top row) and gp340 expression (bottom row) in columnar epithelia of the colon/rectum. Serial 5um sections were stained by IHC for gp340 and CD16, as described in Materials and Methods. The three CD16/gp340 pairs of images represent samples from three different subjects, and each pair is from the same site of the specimen. Gp340 and CD16 are stained brown (some examples marked with arrows), and cell nuclei are blue. Note intraepithelial CD16^+^ cells and brown granular staining of gp340 throughout the non-mucous columnar cells at the same sites.
(EPS)

S4 Fig Examples of the distribution of CD4^+^ cells and gp340 expression in ectocervical stratified squamous (A, B) and endocervical columnar (C, D) epithelia. Serial 5um sections were stained by IHC for gp340 and CD16, as described in Materials and Methods, and images from the same area of the specimens are shown. Gp340 and CD16 are stained brown (some examples marked with arrows), and cell nuclei are blue. Note the presence of CD4^+^ cells the brown granular pattern of gp340 staining throughout the ectocervical squamous cells of the spinous layer above the basal undifferentiated keratinocytes, as well as in the majority of the endocervical columnar epithelial cells. Also note the distribution of CD4^+^ cells among the columnar epithelial cells at the same location.
(EPS)

S5 Fig Interface between the epithelium and the underlying lamina propria is identifiable on the basis of morphology. Sections of colon/rectum (A and B) and of endocervix (C and D) were stained by standard H&E (top panels) or by IHC for CD16. The interface between the epithelium and the lamina propria is indicated with black arrows. CD16^+^ cells are stained brown. Note many intra-epithelial CD16^+^ cells (above the basement membrane).
(EPS)
